# Tempeh-type fermentation kinetics of tarwi (*Lupinus mutabilis*) and red quinoa (*Chenopodium quinoa*) with *Rhizopus oligosporus*: effects on texture, metabolite profile and nutritional potential

**DOI:** 10.3389/fnut.2026.1904999

**Published:** 2026-08-27

**Authors:** Rafael Alarcón, Bernardo O. Yépez Silva-Santisteban, Elizabeth Villanueva-Quejia, Jasmin Raita, Ville Koistinen, Kati Hanhineva, Ritva Repo-Carrasco-Valencia

**Affiliations:** 1 Centro de Investigación e Innovación en Productos Derivados de Cultivos Andinos CIINCA, Universidad Nacional Agraria La Molina, Lima, Peru; 2 Facultad de Industrias Alimentarias, Universidad Nacional Agraria La Molina, Lima, Peru; 3 Food Sciences Unit, University of Turku, Turku, Finland; 4 Institute of Public Health and Clinical Nutrition, University of Eastern Finland, Kuopio, Finland

**Keywords:** kinetics, quinoa, *Rhizopus oligosporus*, tarwi, tempeh

## Abstract

Tempeh is a solid-state fermented food traditionally produced from soybeans using fungi of the genus *Rhizopus*, which modify the nutritional and functional properties of the substrate. The search for alternative raw materials has increased interest in underutilized Andean crops such as tarwi (*Lupinus mutabilis* cv. Cholo Fuerte) and quinoa (*Chenopodium quinoa* cv. Pasankalla), recognized for their high nutritional value and potential for sustainable food development. This study investigated the growth kinetics of *Rhizopus oligosporus* during nine days of tempeh-type fermentation of tarwi and quinoa, indirectly evaluated enzyme production through enzymatic activity, and characterized associated changes in texture and metabolomic profiles. Biomass growth was best described by the Gompertz model, whereas enzyme production followed the Luedeking–Piret model, indicating predominantly growth-associated behavior. Tarwi tempeh showed a better fit to both models and greater proteolytic activity, whereas quinoa tempeh exhibited poorer model fitting and predominantly amylolytic activity. Textural properties were influenced by fungal growth and enzymatic activity, with changes in hardness, cohesiveness, springiness, and fragility; optimal characteristics were observed between 48 and 72 h of fermentation. Metabolomic analysis identified 448 metabolites, revealing clear substrate-dependent clustering. Quinoa tempeh was characterized by metabolites related to carbohydrate metabolism, nucleotide turnover, and nucleoside derivatives, indicating active fermentative metabolism. In contrast, tarwi tempeh showed a metabolomic profile dominated by amino acid-related compounds, reflecting intensified proteolysis and amino acid metabolism. These findings highlight the key role of substrate composition in modulating fungal growth, enzyme activity, textural properties, and metabolomic profiles during tempeh fermentation.

## 1 Introduction

Fermentation is one of the earliest food processing techniques and involves the use of microorganisms, including bacteria, molds, and yeasts, to transform food matrices (1). As a significant biotechnological process, fermentation enhances nutritional quality, food safety, and the production of bioactive compounds through microbial activity (2). During fermentation, microbial and endogenous enzymatic activities promote hydrolytic transformations that degrade antinutritional factors while improving sensory attributes and nutritional quality (3). Microbial metabolism not only contributes to food preservation but also drives the synthesis and release of health-promoting metabolites, such as vitamins, bioactive peptides, antioxidants, and short-chain fatty acids (4). In this context, fungal fermentations have emerged as a particularly effective strategy for the valorization of protein-rich plant matrices, owing to their strong enzymatic capacity to modify complex macromolecules and enhance protein availability (5).

Solid-state fermentation (SSF) is an effective strategy for enhancing the nutritional value of pulses through microbial biotransformation. The filamentous fungus genus *Rhizopus*, generally recognized as safe (GRAS) by the U.S. Food and Drug Administration (FDA), is widely employed in SSF processes, particularly in Asia, for the production of fermented foods such as tempeh (6). Tempeh is a traditional fermented pulse product originating from Southeast Asia, produced through the growth of *Rhizopus* spp. on cooked soybeans, which results in a compact, cake-like structure. In SSF systems, fungal growth occurs on moist solid substrates with low free water content, which facilitates efficient oxygen diffusion and close substrate–mycelium contact, conditions essential for optimal metabolic activity (7). During fermentation, *Rhizopus* spp. secrete a broad range of enzymes, including extracellular proteases and *α*-amylases, which drive the formation of various metabolites, such as amino acids and simpler carbohydrates (6, 8). These enzymatic activities hydrolyze complex macromolecules, thereby improving protein digestibility and nutrient bioavailability (9).

In recent years, increasing attention has been directed toward the use of underutilized crops of regional importance as alternatives to soybeans in the production of fermented foods. Among these, Andean crops such as quinoa (*Chenopodium quinoa*) stand out not only for their distinctive nutritional composition but also for their cultural relevance in high Andean regions and their adaptability to harsh agroclimatic conditions. Quinoa is a nutrient-dense pseudocereal rich in minerals, dietary fiber, and bioactive compounds, with a protein content and amino acid profile comparable to that of casein (10). Similarly, tarwi (*Lupinus mutabilis*), an indigenous Andean legume from South America, is characterized by its high protein content and a favorable amino acid and lipid profile (11). Despite their high nutritional potential, both crops present technological and dietary challenges due to the presence of antinutritional compounds, including saponins, phytates, tannins, bitter quinolizidine alkaloids, and enzyme inhibitors, which can negatively affect mineral bioavailability, sensory attributes, and digestibility in food formulations (12). Furthermore, tarwi remains an underutilized crop despite its high nutritional and agronomic potential. Ecuador, Peru, and Bolivia are the main producers in South America, with estimated annual per capita consumption of approximately 4.0, 0.5, and 0.2 kg, respectively (13). In Peru, national tarwi production declined from 16,481 tons in 2018 to 15,084 tons in 2022 (14). Although quinoa is more widely recognized and commercially utilized than tarwi, its production also declined in Peru, from 114,200 tons in 2022 to 72,800 tons in 2023 (15). Therefore, developing value-added foods from these crops represents an important strategy to promote their utilization and commercialization. In this context, solid-state fermentation has been proposed as an effective approach to reduce antinutritional factors while enhancing nutrient bioavailability, sensory quality, and functional properties (16).

Optimizing tempeh-type fermentation processes requires a thorough understanding of fermentation kinetics, as microbial growth and enzymes production evolve continuously throughout fermentation time and are strongly influenced by both the inoculum characteristics and the composition of the substrate, the enzyme production can be indirectly assessed through enzymatic activity, since enzyme activity is directly proportional to the amount of active enzyme present in a sample (17). Studies employing *Rhizopus* across different substrates have reported substantial variations in enzymes production, metabolite production and texture development, indicating that substrate composition plays a key role in modulating fermentation dynamics and determining final product quality (18–21). Despite these advances, there is still limited understanding of how *R. oligosporus* fermentation kinetics interact with substrate composition to modulate enzyme production, influence on texture, and metabolite profiles in alternative tempeh matrices such as tarwi (*Lupinus mutabilis*) and red quinoa (*Chenopodium quinoa*).

The objective of this study was to investigate the growth kinetics and enzyme production of *Rhizopus oligosporus* during tempeh-type fermentation of tarwi (*Lupinus mutabilis*, variety Cholo Fuerte) and quinoa (*Chenopodium quinoa*, variety Pasankalla) over 9 days and to evaluate the associated changes in texture, metabolomic profiles, and potential nutritional properties.

## 2 Materials and methods

### 2.1 Raw materials and reagents

The Cholo Fuerte variety of tarwi was obtained from the Cereals and Legumes Program of the National Agrarian University La Molina, while the Pasankalla variety of mechanically scarified quinoa was obtained from the Native Cereals and Grains Program of the same institution. The *Rhizopus oligosporus* starter strain was purchased from Natural Asia Products. All reagents were purchased from Sigma Aldrich at analytical grade.

### 2.2 Solid-state fermentation (SSF) of tempeh

Tarwi grains were debittered to remove alkaloids following a previously reported method (22) with minor modifications. A total of 500 g of tarwi were soaked in 5 L of water at room temperature for 24 h. The soaking water was discarded, and the grains were cooked in 2.5 L of water for 30 min, then drained and cooked again in an additional 2.5 L of water for 45 min. The grains were subsequently soaked in 4 L of water for 5 days, with the soaking water replaced every 8 h. After debittering, the grains were manually hulled and cooked for an additional 30 min. Quinoa grains were washed with abundant water for 30 min and cooked for 8 min. At the end of each cooking step, vinegar was added until the pH reached between 3.5 and 4.5. The water was then drained, and the grains were gently heated to remove residual surface moisture.

Tarwi and quinoa were fermented separately, fermentation began by inoculating commercial starter of *Rhizopus oligosporus* in a proportion of 1 g of starter per 100 g of substrate. The inoculum was evenly distributed and mixed for 3 min. Subsequently, 45 g of the inoculated substrate were transferred into Ziploc bags (3×3 cm^2^) perforated on both sides (3 × 3 holes) using a disinfected pin. Incubation was conducted at 30 °C for 9 days. The moisture content of both tarwi and quinoa grains was to approximately 60% prior to fermentation. Samples were collected at random at 0, 1, 2, 3, 4, 7, 8, and 9 days of fermentation. Fresh samples were used for texture measurement and then were dried at 45 °C for 24 h, ground, and stored in a freezer until further analysis.

### 2.3 Biomass of *Rhizopus*

The biomass was calculated indirectly by determining the glucosamine content following a previously reported method (23). The analysis was conducted under a fume hood by weighing 0.5 g of sample into screw-cap test tubes and adding 5 mL of 6 N hydrochloric acid. The samples were hydrolyzed in a water bath at 100 °C for 2 h. After cooling to room temperature, the hydrolysates were filtered, then 1 mL of the filtrate was neutralized with 6 N sodium hydroxide and adjusted to a final volume of 10 mL.

Subsequently, 1 mL of acetylacetone solution in 0.5 N sodium carbonate was added to 1 mL of the neutralized solution. The mixture was heated at 100 °C for 20 min in a water bath and then cooled. Thereafter, 6 mL of absolute ethanol and 1 mL of Ehrlich’s reagent, p-dimethylaminobenzaldehyde prepared in acidic medium, were added. The blank was prepared using 1 mL of distilled water. The mixtures were vortexed and incubated at 65 °C for 10 min in a water bath, followed by cooling to room temperature. Absorbance was measured at 530 nm using a UV–Vis spectrophotometer (1900i, Shimadzu, Kioto, Japan). Glucosamine concentration was determined using a calibration curve ranging from 20 to 160 mg/L, prepared from a 1 g/L stock solution. Biomass content was expressed as grams of biomass per gram of dry sample, applying a conversion factor of 12 g fungal biomass per g glucosamine (24).

### 2.4 Amylase and protease activities

The enzymatic extracts were prepared following a previously reported method, with minor modifications (25). 1 g sample was mixed with 10 mL citrate buffer (pH 5.5) in a flask and then shaked at 25 °C for 30 min to extract enzymes (ZWY-200D, Labwit, Melbourne, Australia). Subsequently, the mixture was centrifuged at 6,500 × g and 4 °C for 15 min (5430R, Eppendorf AG, Hamburg, Germany). The supernatant obtained was used as a crude enzyme solution.

Amylase and protease activities were determined according to a previously reported method (21), with modifications. For amylase activity, 0.25 mL of enzyme extract was mixed with 0.25 mL of water and incubated with 0.5 mL of 1% soluble starch at 37 ° C for 5 min. After adding 0.5 mL of DNS reagent (prepared with 3,5-dinitrosalicylic acid, sodium hydroxide, and sodium potassium tartrate in a 1:2:30 w/v ratio), the mixture was incubated at 95 °C for 15 min, cooled, and diluted with 9 mL of water. Absorbance was measured at 540 nm using a spectrometer (1900i, Shimadzu, Kioto, Japan). A maltose standard curve (100–1800 μg/mL) was prepared from a 2 mg/mL stock solution and one unit of amylase activity was defined as the amount of enzyme required to release 1 μmol/min of reducing sugar. For protease activity, 0.5 mL of enzyme extract was mixed with 0.5 mL of water, and then 1 mL of 1% casein was incubated at 37 °C for 30 min. After adding 2 mL of 10% TCA to terminate the reaction, the mixture was centrifuged at 6,500 × g and 25 °C for 15 min. 1 mL of supernatant was mixed with 2.5 mL of sodium carbonate, and 0.5 mL of 50% Folin–Ciocalteu phenol reagent was added to each tube. The resulting mixture was thoroughly mixed and incubated at 37 °C for 30 min. Absorbance was read at 660 nm. A tyrosine standard curve (25–250 μg/mL) was prepared from a 500 μg/mL stock solution and one unit of protease activity was defined as the amount of enzyme required to release 1 *μ*mol/min of tyrosine.

### 2.5 Growth and enzyme production modelling

Biomass growth was modeled using the Gompertz and Logistic equations (26) (Equations 1, 2), whereas enzyme production was described using the Luedeking–Piret equation (27) (Equation 3).


$$X\;(t)=X_{\max}\cdot \exp\;(-b\cdot \exp(-\mu \cdot t))$$ (1)

$$X\;(t)=\frac{X_{\max}}{1+(\;\frac{X_{\max}-X_{0}}{X_{0}})\exp(-\mathrm{kt})}$$ (2)

$$P\;(x)=P_{0}+\alpha (X-X_{0})+\frac{\beta X_{\max}}{\mu }\ln(\;\frac{X_{\max}-X_{0}}{X_{\max}-X})$$ (3)

Where X(t) is the biomass concentration at time (*t*), X_max_ is the maximum biomass concentration, X_0_ is the initial biomass concentration, P(X) is the enzymatic activity as a function of biomass concentration, and P0 is the initial enzymatic activity. The parameters µ and k are the growth rate constants of the Gompertz and Logistic models, respectively, while b is the dimensionless shape parameter of the Gompertz model. The parameters *α* and *β* are the growth-associated and non-growth-associated Luedeking–Piret coefficients, respectively, and t is the fermentation time.

Model performance was evaluated using statistical parameters, including the coefficient of determination (*R*^2^), chi-square (*χ*^2^), root mean square error (RMSE), and residual sum of squares (RSS), to identify the most appropriate model for describing fermentation kinetics, following a previously reported approach. Model parameters were estimated using the Solver function by minimizing the RSS values.

### 2.6 Texture profile analysis (TPA)

TPA was performed on same-sized tempeh samples that was 20 mm × 20 mm × 10 mm (length×width×height) using a texture analyzer (3,365, Instron, Massachusetts, USA) with a 50 mm cylindrical probe. The testing conditions were as follows: speed of 1 mm/s, compressed to 50% of their original height, and loadcell of 10 Kgf. The parameters assessed using the two-bite compression test included hardness, fragility, springiness, cohesiveness, gumminess, and chewiness (21).

### 2.7 Metabolomic analysis

#### 2.7.1 Sample preparation

Approximately 5 mg of dried sample was added into a 15 mL homogenizer tube (Precellys CK28, Bertin Technologies SAS, Montigny-le-Bretonneux, France) and homogenized (Precellys Evolution, Bertin Technologies SAS) with the following settings: 4500 rpm, room temperature, 2 × 30 s cycle with a 20-s break. Approximately 100 mg of the homogenized sample (with the exact weight measured) was then inserted into an Eppendorf tube. The extraction solvent, which consisted of 80% v/v LC–MS-grade methanol and 20% v/v of ultra-pure water, was added onto the samples in the Eppendorf tubes in a ratio of 600 μL per 100 mg of sample. The samples were then vortexed for 10 s in room temperature and let stay in room temperature for 15 min before another vortexing of 10 s (in the same order). The samples were centrifuged (VWR Mega Star 600R) for 10 min at 17000 × g and +4 °C. The supernatant was collected with a 1 mL syringe and filtered through a syringe filter (0.2 μm PTFE) into brown HPLC vials with inserts. Pooled quality control (QC) samples were prepared by pooling 20 μL of each sample and then filtering through the syringe filter into a separate vial. Extraction blank samples were prepared by adding the extraction solvent to empty Eppendorf tubes and treating them the same way as the study samples thereafter. Sample TQ01 was prepared in triplicate to control for instrument variability.

#### 2.7.2 LC–MS/MS analysis

The LC–MS analysis was performed as previously described (28). In brief, the samples were analyzed using liquid chromatography quadrupole time-of-flight mass spectrometry UHPLC–QTOF–MS system (Agilent Technologies), which consisted of a 1,290 LC system, a Jetstream electrospray ionization (ESI) source, and a 6,540 UHD QTOF mass spectrometer. The samples were separated using reversed-phase (RP) chromatography (Zorbax Eclipse XDB-C18, particle size 1.8 μm, 2.1 × 100 mm; Agilent Technologies). The elution solvents were water (A) and HPLC grade methanol (B), both containing formic acid 0.1% v/v. The gradient was as follows for the ratio of solvent B: 0–10 min: 2 → 100% B; 10–14.5 min: 100% B; 14.5–14.51 min: 100 → 2% B; 14.51–16.5 min: 2% B. Data was acquired with both positive and negative polarity. Quality controls were injected at the beginning and at the end of the MS run and after every ten injections. Automatic data-dependent MS/MS analysis was performed on one sample representing each sample type. The sample tray was kept at +4 °C during the analysis. The samples of quinoa and tarwi tempeh were separated in the sequence but randomized within each sample type.

#### 2.7.3 LC–MS data analysis

The raw data from the LC–MS instrument was processed in MS-DIAL version 4.9.221218 For the peak picking, MS1 tolerance was set to 0.01 Da, MS2 tolerance 0.025 Da, m/z range 50–1,500 (small molecules), minimum peak amplitude 2000 signal counts, and mass slice width 0.1 Da. Peak smoothing was performed using linear weighted moving average; the smoothing level was 3 scans and minimum peak width 5 scans. The adduct ions were selected as follows: [M + H]+, [M + NH4]+, [M + Na]+, [M + CH3OH + H]+, [M + K]+, [2 M + H] + for the positive mode and [M − H]−, [M − H2O − H]−, [M + Cl]−, [M + FA − H]−, [2 M − H] − for the negative mode. For the peak alignment, m/z tolerance was 0.015 Da and retention time tolerance 0.05 min. Gap filling by compulsion function was utilized to forcibly detect peak areas *ad hoc* within 5 data points even if no local peak maxima were detected. The peaks of the annotated compounds were curated manually if the automated peak picking had resulted in integration errors. After aligning the detected signals across all samples, the remaining 65,637 individual molecular features, including those originating from the positively and negatively ionized molecules, were compiled into an Excel datasheet for further data analysis and compound annotation.

Principal component analysis (PCA) was performed for the 7,102 most abundant molecular features (average peak area >200,000 signal counts) in RStudio v. 2025.09.1 utilizing in-house scripts, the ggplot function in R package ggplot2, and biplot scaling based on Euclidean distance matrix. Quality control samples were plotted in the PCA to assess potential signal drift during the LC–MS run. Shannon’s diversity index for the phytochemical abundances was calculated in R Studio using vegan package version 2.5–645 (29). The heat map was generated in RStudio using relative metabolite abundances. Group mean signal intensities were normalized by metabolite-wise z-score scaling according to the formula z = (x− x̄_row_)/SD_row_, where each metabolite was centered and scaled across samples. Hierarchical clustering was applied to both metabolites and sample groups to visualize similarities in metabolomic profiles.

#### 2.7.4 Annotation of compounds

The molecular features were annotated in a semi-targeted manner, utilizing literature on previously detected phytochemicals in cereals. A NIST compatible MSP database file, containing, e.g., MassBank, GNPS (Global Natural Products Social Molecular Networking), RIKEN spectral databases, and our in-house reference standard library, was utilized in MS-DIAL for additional annotations and mass spectral comparison. The reliability of each annotation was assessed according to the Metabolomics Standards Initiative (30): level 1 was given to true identifications confirmed with a reference standard analyzed with the same instrument and LC–MS method; level 2 included putative annotations based on the exact mass, calculated molecular formula, and MS/MS fragmentation spectra; level 3 was used as the classification for putative characterization of compound class, based on characteristic MS/MS fragmentation pattern and additional physicochemical properties, such as retention time.

### 2.8 Statistical analysis

The results were expressed as the mean ± standard deviation. All measurements were determined in triplicate. The ANOVA was used to analyses the acquired data at a 95% significance level using Minitab 18.0 Software (Minitab Inc., State College, Palo Alto, CA, USA).

## 3 Results

### 3.1 Biomass

The biomass values for tarwi and quinoa tempeh are presented in Table 1. In tarwi tempeh, biomass increased progressively throughout fermentation, reaching a maximum of 17.62 ± 1.34 g/g at 216 h. In contrast, quinoa tempeh exhibited rapid biomass accumulation during the first 24 h, followed by a statistically stable phase, with a subsequent increase observed at 216 h (12.33 ± 0.81 g/g).

**Table 1:** Biomass content and enzymatic activity of tempeh.

Parameter	Tempeh	Time (h)							
		0	24	48	72	96	168	192	216
Biomass (g/g sample)	Tarwi	3.72 ± 0.32^e^	6.82 ± 0.21^d^	8.51 ± 0.32^cd^	9.03 ± 1.57^cd^	11.31 ± 1.59^bc^	12.36 ± 1.38^b^	14.50 ± 0.94^b^	17.62 ± 1.34^a^
	Quinoa	2.07 ± 0.10^d^	8.00 ± 0.63^c^	8.00 ± 0.84^c^	9.20 ± 0.27^c^	9.13 ± 1.03^c^	9.88 ± 1.43^bc^	11.60 ± 0.57^ab^	12.33 ± 0.81^a^
Amylases activity (U/g)	Tarwi	2.14 ± 0.10^e^	4.67 ± 0.73^d^	4.43 ± 0.13^d^	11.73 ± 0.23^c^	10.45 ± 0.05^c^	16.71 ± 0.51^a^	10.78 ± 0.43^c^	13.83 ± 0.53^b^
	Quinoa	10.58 ± 0.20^ab^	10.97 ± 0.58^a^	13.79 ± 0.63^a^	10.93 ± 0.15^a^	11.19 ± 0.58^a^	12.45 ± 2.17^a^	6.38 ± 2.18^bc^	4.40 ± 0.07^c^
Proeases activity (U/g)	Tarwi	0.16 ± 0.02^f^	0.27 ± 0.01^e^	0.39 ± 0.02^d^	0.53 ± 0.00^c^	0.60 ± 0.02^b^	0.86 ± 0.02^a^	0.85 ± 0.04^a^	0.90 ± 0.01^a^
	Quinoa	0.01 ± 0.00^g^	0.04 ± 0.00^f^	0.14 ± 0.01^cd^	0.11 ± 0.00^e^	0.16 ± 0.01^c^	0.21 ± 0.01^b^	0.13 ± 0.00^de^	0.25 ± 0.01^a^

Results are expressed as mean ± SD. a, b, c, d, e values in the same rows with different letters differ significantly (*p* < 0.05).

### 3.2 Enzymatic activity

The enzymatic activities of amylases and proteases are presented in Table 1. In tarwi tempeh, amylase activity increased progressively, reaching a maximum of 16.71 ± 0.51 U/g at 168 h, followed by a decline. In contrast, amylase activity in quinoa tempeh remained statistically unchanged up to 168 h and subsequently decreased to 4.40 ± 0.07 U/g at 216 h.

Regarding protease activity, tarwi tempeh showed a continuous increase throughout fermentation, rising from 0.16 ± 0.02 U/g to 0.90 ± 0.01 U/g at 216 h. In contrast, quinoa tempeh also exhibited an increasing trend, although at a slower rate, with values ranging from 0.01 ± 0.00 U/g to 0.25 ± 0.01 U/g over the same fermentation period.

### 3.3 Growth and enzyme production modelling

Tables 2, 3 presents the parameters of the fitted models (b, *μ*, *α*, *β*) and statistical indicators (*R*^2^, *χ*^2^, and RMSE) obtained for the Gompertz and Logistic models fitted to biomass growth in tarwi and quinoa tempeh, as well as for the Luedeking–Piret model applied to enzyme production. Overall, the Gompertz model exhibited a higher coefficient of determination (*R*^2^ = 0.77–0.90) compared with the Logistic model (*R*^2^ = 0.73–0.87) in both raw materials. In addition, the Gompertz model generally showed lower *χ*^2^, RMSE, and RSS values, indicating a better fit to the experimental data. For tarwi tempeh, the Gompertz model parameters were 1.387 and 0.01 h^−1^ for b and *μ*, respectively, and for quinoa tempeh, they were 1.173 and 0.013 h^−1^ for b and μ, respectively.

**Table 2:** Results of the growth model adjustment.

Model parameters and model fit statistics	Gompertz model		Logistic model	
	Tarwi	Quinoa	Tarwi	Quinoa
X_max_ (g/g)	18.420	13.240	18.420	13.240
b	1.387	1.173	–	–
μ (h^−1^)/ k (h^−1^)*	0.010	0.013	0.017	0.036
R^2^	0.902	0.772	0.878	0.731
χ2	3.931	6.931	9.437	19.172
RMSE	45.2	43.1	78.2	121.0

*μ (h^−1^) for the Gompertz model and k (h^−1^) K for the Logistics model.

**Table 3:** Results of the adjustment of the production model (Luedeking–Piret).

Model parameters and model fit statistics	Amylases		Proteases	
	Tarwi	Quinoa	Tarwi	Quinoa
X_0_ (g/g)	3.40	1.97	3.40	1.97
X_max_ (g/g)	18.42	13.24	18.42	13.24
μ (h^−1^)	0.010	0.013	0.010	0.013
P_0_ (U/g)	3.4	10.44	0.15	0.01
*α* (U/g)	0.88	0.229	0.054	0.016
*β* (U/g.h)	10^−5^	10^−5^	3×10^−5^	10^−5^
*R* ^2^	0.79	0.05	0.98	0.74
*χ* ^2^	12.14	2.09	0.08	0.26
RMSE	101.31	24.74	0.03	0.03

For the analysis of enzyme production, the Luedeking–Piret model was applied using the biomass parameters previously obtained from the Gompertz model. In quinoa, the model was fitted using data up to 168 h, since the higher variability observed after this time prevented an adequate fit.

**Figure 1:**
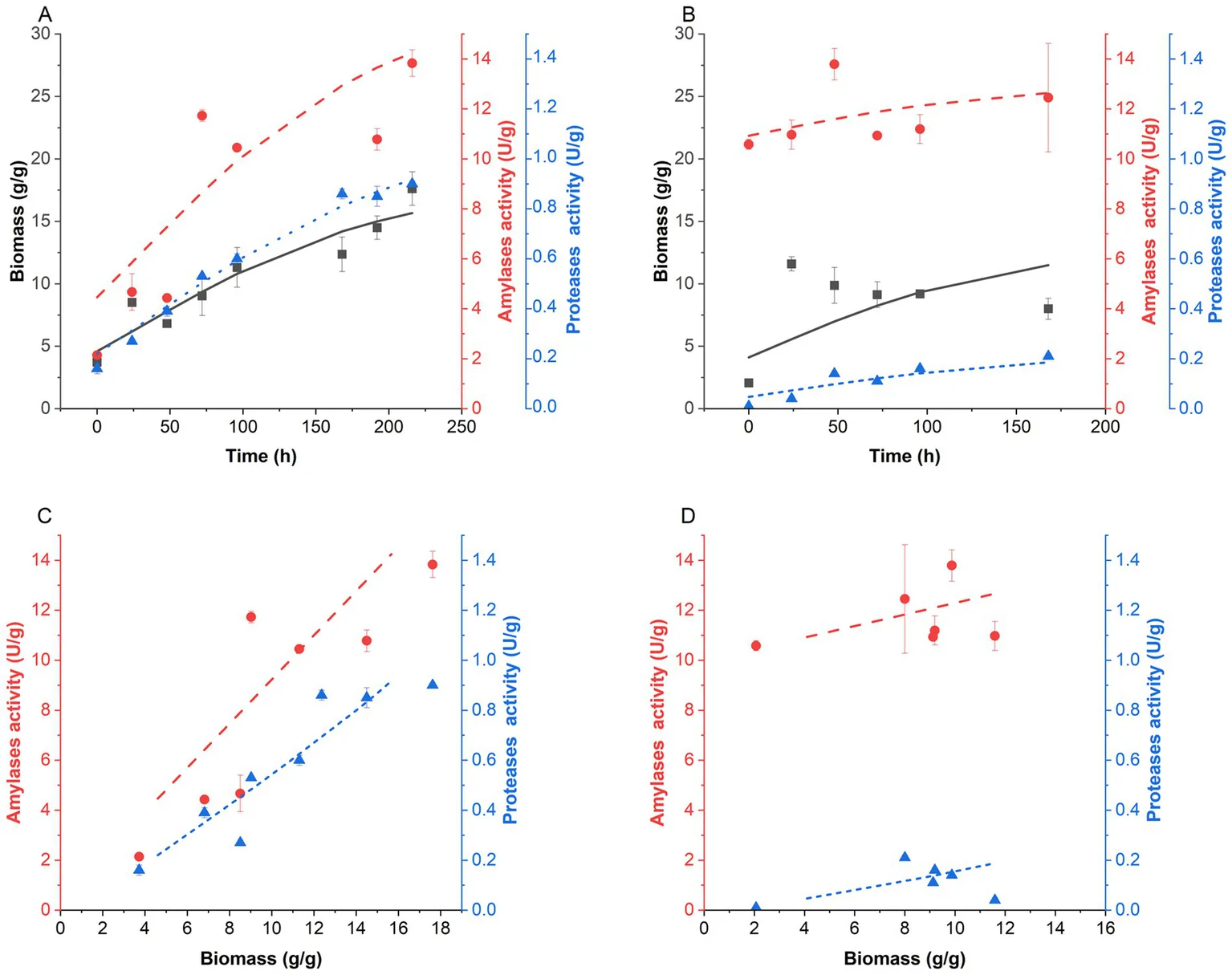
Growth and enzymes production kinetics. **(A,C)** Tarwi tempeh and **(B,D)** quinoa tempeh. Experimental data of biomass (■), amylase (●), and protease (▲). Predicting kinetic model: Gompertz model for biomass (˗˗˗) and Gompertz and Luedeking-Piret models for amylase (˗ ˗ ˗), and protease (˖˖˖˖˖).

Figure 1 shows the biomass adjusted by the Gompertz model and the enzymes production adjusted by the Luedeking–Piret model. An increase in biomass and enzymatic activity was observed as fermentation progressed (Figures 1A,B). Furthermore, higher biomass levels were associated with increased amylase and protease activities (Figures 1C,D).

### 3.4 Texture profile analysis (TPA)

**Figure 2:**
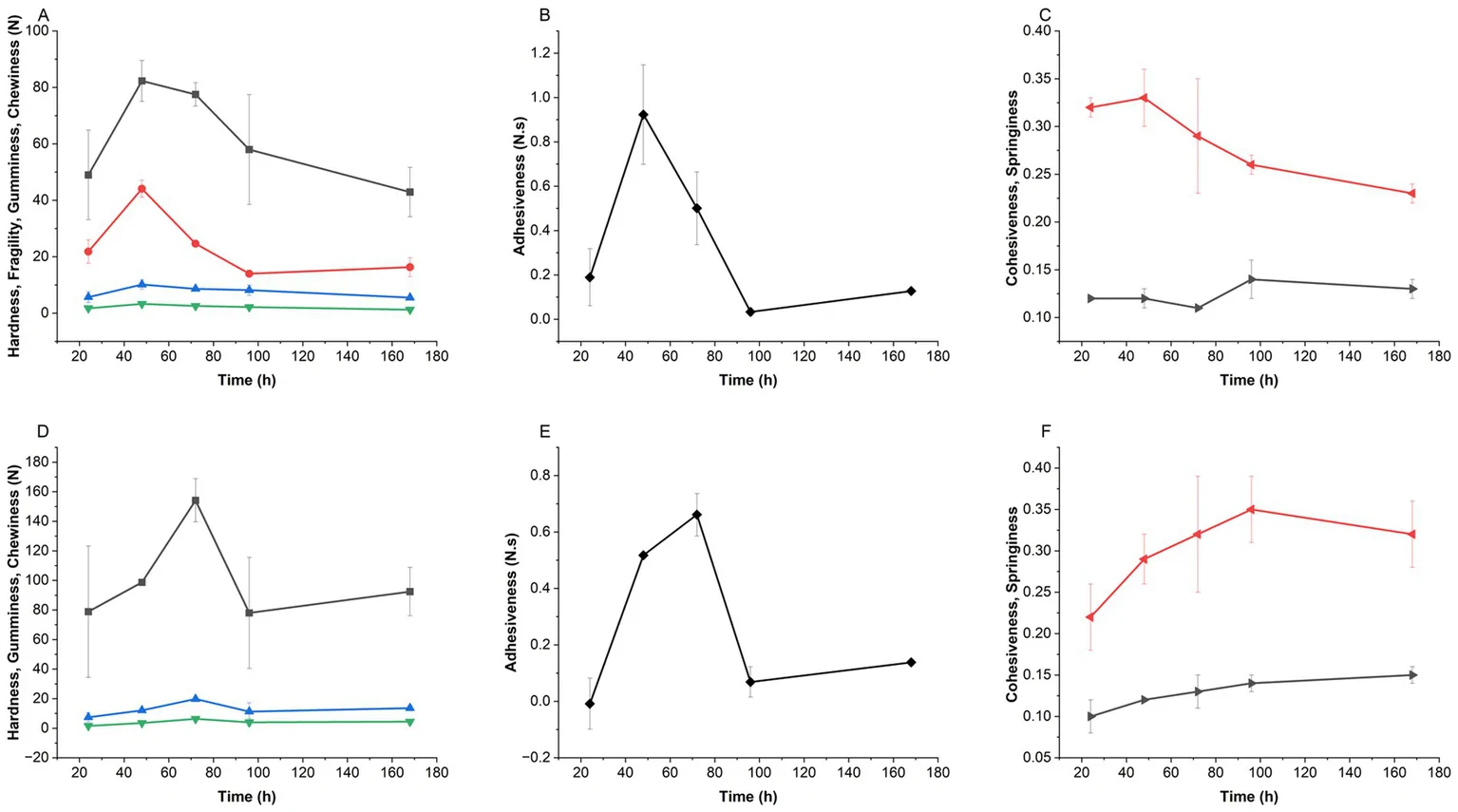
**(A)** Tarwi tempeh and **(D)** quinoa tempeh, hardness (■), fragility (●), gumminess (▲), chewiness (▼). **(B)** Tarwi tempeh and **(E)** quinoa tempeh, adhesiveness (♦). **(C)** Tarwi tempeh and **(F)** quinoa tempeh, cohesiveness (►), springiness (◄).

Table 4 and Figure 2 presents the texture profile analysis (TPA) of quinoa and tarwi tempeh, including hardness, fracturability, adhesiveness, cohesiveness, springiness, gumminess, and chewiness. Texture analysis was not conducted at 0 h because the absence of mycelial growth prevented the formation of a compact structure suitable for testing. In addition, samples at 192 and 216 h were excluded due to exudate formation and water loss during fermentation.

**Table 4:** The results of texture profile analysis.

Tempeh	Fermentation time (h)	Hardness (N)	Fragility (N)	Adhesiveness (N.s)	Cohesiveness	Springiness	Gumminess (N)	Chewiness (N)
Quinua	24	78.85 ± 44.49^b^	Nd	−0.01 ± 0.09^a^	0.10 ± 0.02^b^	0.22 ± 0.04^b^	7.41 ± 3.08^b^	1.56 ± 0.40^b^
	48	98.67 ± 0.17^ab^	Nd	0.52 ± 0.01^a^	0.12 ± 0.00^ab^	0.29 ± 0.03^ab^	12.14 ± 0.17^ab^	3.52 ± 0.33^ab^
	72	154.14 ± 14.60^a^	Nd	0.66 ± 0.75^a^	0.13 ± 0.02^ab^	0.32 ± 0.07^ab^	19.76 ± 1.35^a^	6.35 ± 1.81^a^
	96	78.00 ± 37.56^b^	Nd	0.07 ± 0.05^a^	0.14 ± 0.01^a^	0.35 ± 0.04^a^	11.25 ± 6.02^ab^	4.02 ± 2.51^ab^
	168	92.45 ± 16.31^ab^	Nd	0.14 ± 0.01^a^	0.15 ± 0.01^a^	0.32 ± 0.04^ab^	13.62 ± 1.89^ab^	4.45 ± 1.22^ab^
Tarwi	24	49.98 ± 15.85^ab^	21.86 ± 4.16^b^	0.19 ± 0.13^bc^	0.12 ± 0.00^ab^	0.32 ± 0.01^a^	5.70 ± 1.94^b^	1.76 ± 0.60^ab^
	48	82.31 ± 7.22^a^	44.08 ± 3.05^a^	0.92 ± 0.22^a^	0.12 ± 0.01^ab^	0.33 ± 0.03^a^	10.15 ± 1.65^a^	3.31 ± 0.27^a^
	72	77.52 ± 4.16^a^	24.63 ± 0.00^b^	0.50 ± 0.16^b^	0.11 ± 0.00^b^	0.29 ± 0.06^ab^	8.64 ± 0.71^ab^	2.56 ± 0.75^ab^
	96	58.01 ± 19.46^ab^	14.02 ± 0.00^c^	0.03 ± 0.02^c^	0.14 ± 0.02^ab^	0.26 ± 0.01^ab^	8.17 ± 1.86^ab^	2.17 ± 0.58^ab^
	168	42.94 ± 8.75^b^	16.32 ± 3.33^c^	0.13 ± 0.00^c^	0.13 ± 0.01^ab^	0.23 ± 0.01^b^	5.52 ± 0.72^b^	1.26 ± 0.24^b^

Nd, Not detected. Results are expressed as mean ± SD. a, b, c values in the same column with different letters differ significantly (*p* < 0.05).

For tarwi tempeh, the highest hardness values were observed on 48 and 72 h (82.31 ± 7.22 and 77.52 ± 4.16 N, respectively). Fragility peaked on 48 h (44.08 ± 3.05 N) and decreased markedly on 96 and 168 h (14.02 ± 0.00 and 16.32 ± 3.33 N). Adhesiveness was also highest on 48 h (0.92 ± 0.22 N·s) and lowest at later stages. Cohesiveness reached its maximum on 96 h (0.38 ± 0.02), while springiness ranged from 0.23 ± 0.01 to 0.33 ± 0.03, with the highest value on 48 h. Gumminess was greatest on 48 h (10.15 ± 1.65 N) and lowest on 24 and 168 h. Chewiness varied between 1.26 ± 0.24 and 3.31 ± 0.27 N, peaking on 48 h (Figures 2A–C).

For quinoa tempeh, samples fermented for 48, 72, and 168 h exhibited the highest hardness values (98.67 ± 0.17, 154.14 ± 14.60, and 92.45 ± 16.31 N, respectively), whereas lower values were observed on 24 and 96 h (78.85 ± 44.49 and 78.00 ± 37.56 N). Adhesiveness did not differ significantly among fermentation times, ranging from 0.01 ± 0.09 to 0.66 ± 0.75 N·s. Cohesiveness was lower on 24 and 48 h (0.10 ± 0.02 and 0.12 ± 0.00) and increased at later stages, reaching values of 0.13 ± 0.02, 0.14 ± 0.01, and 0.15 ± 0.01 on 72, 96, and 168 h, respectively. Springiness ranged from 0.22 ± 0.04 to 0.35 ± 0.04, with 24 h showing significantly lower values. Gumminess was highest on 72 h (19.76 ± 1.35 N) and lowest on 24 h (7.41 ± 3.08 N), while chewiness varied from 1.56 ± 0.40 to 6.35 ± 1.81 N, reaching its maximum on 72 h (Figures 2D–F).

### 3.5 Metabolomics analysis

**Figure 3:**
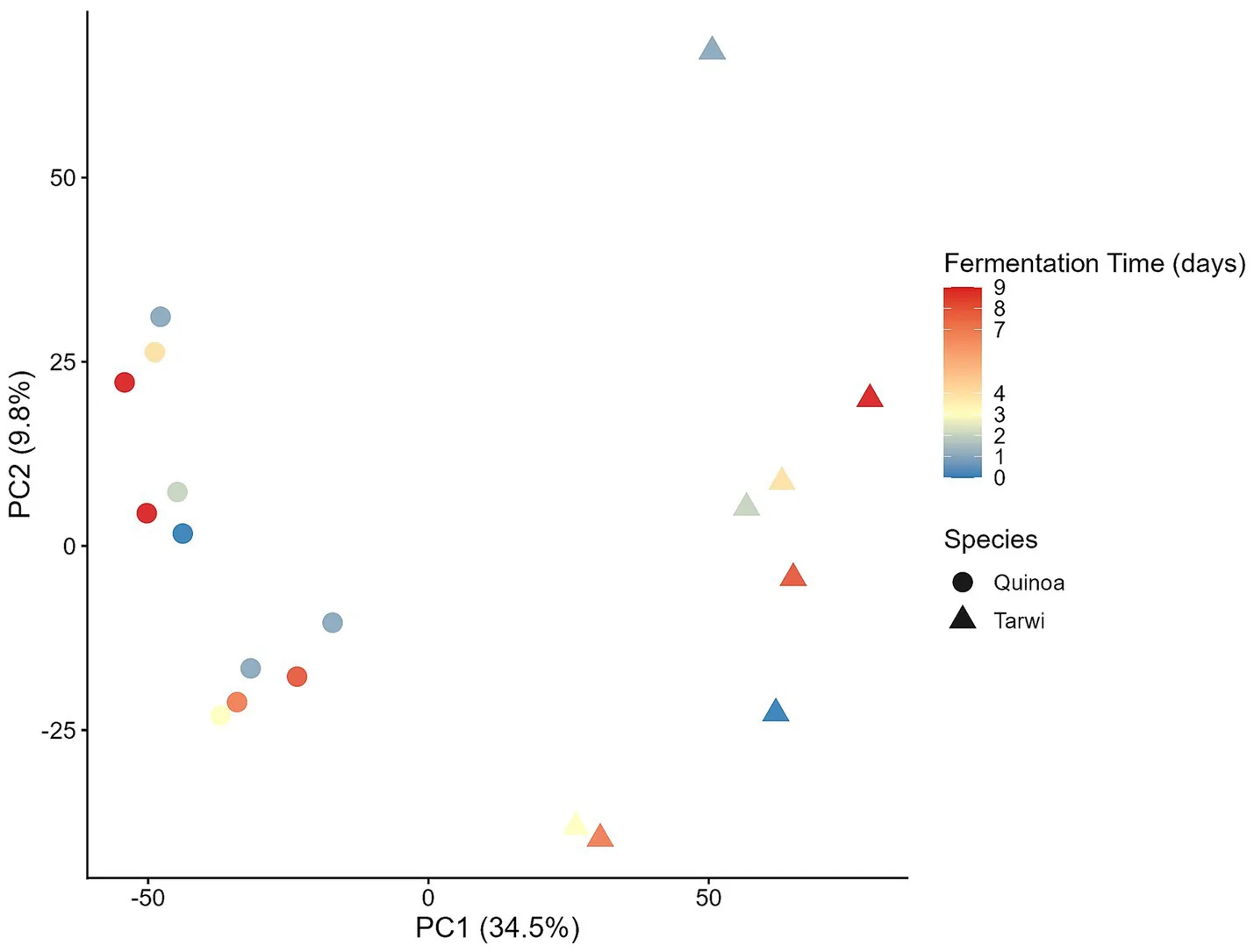
Principal component analysis (PCA) of the most abundant molecular features (*n* = 7,102; average signal intensity >200,000) across all samples. The plot shows the first two principal components, which capture the largest proportion of variance in the dataset. PC1 (34.5%) mainly reflects differences between sample types (quinoa vs. tarwi), while PC2 (9.8%) is associated with fermentation time.

A principal component analysis (PCA) was performed using the 7,102 most abundant molecular features (aligned signals detected by the LC–MS instrument; see Materials and Methods) from both ionization modes to explore and visualize differences in metabolite profiles among samples (Figure 3). The PCA displays the first two orthogonal principal components, PC1 and PC2, which explain 34.5 and 9.8% of the total variance, respectively. PC1 primarily separates samples according to species, revealing a clear distinction between quinoa and tarwi. In contrast, PC2 is mainly associated with fermentation time, with the greatest separation observed between early (day 1) and late (day 9) fermentation stages.

**Figure 4:**
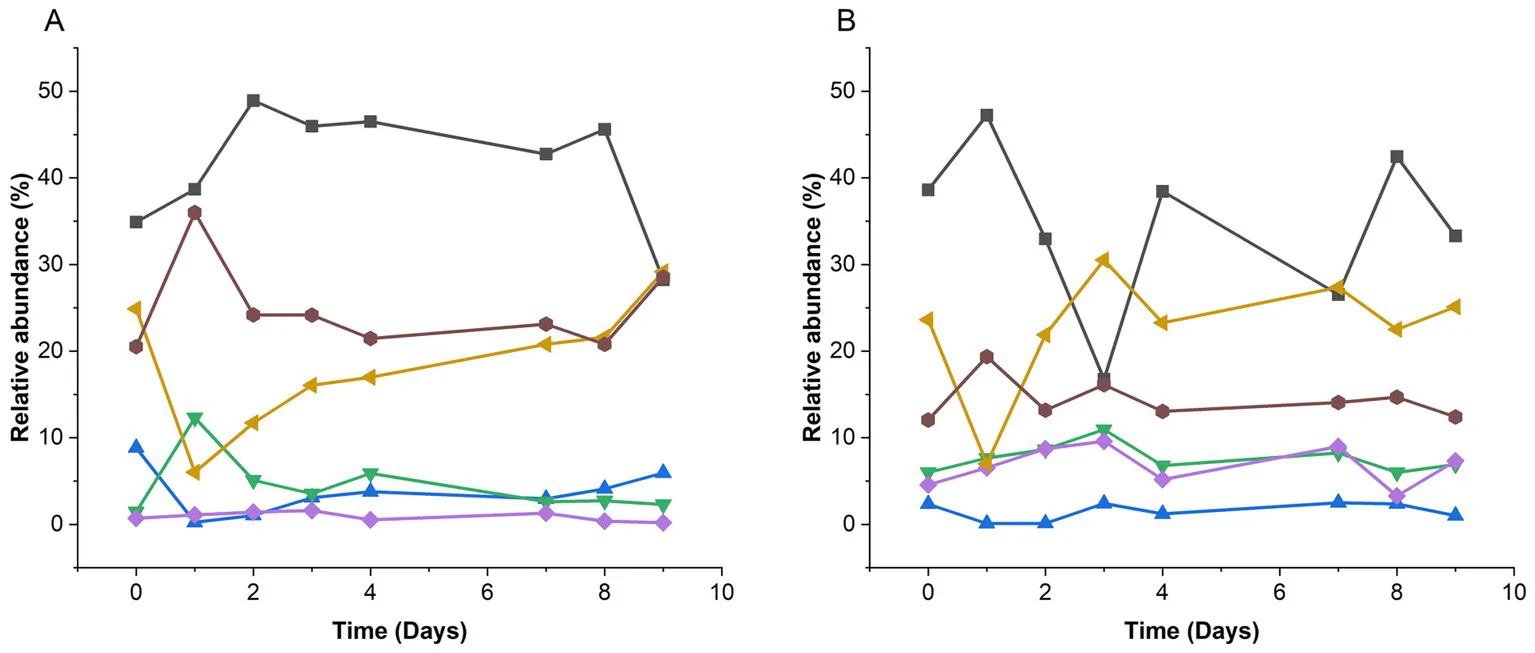
Relative abundance of metabolite groups at different fermentation times, expressed as cumulative signal intensity. **(A)** Tarwi tempeh and **(B)** Quinoa tempeh. Amino acids and derivatives (■), Hydroxy acid (▲), Lipid-PUFA (▼), Nucleosides and derivatives (♦), Organic acids and derivatives (◄), Others (●).

Metabolites belonging to diverse chemical classes were identified, with the most abundant groups including amino acids and derivatives, Choline, fatty acid and derivatives, hydroxy acids, lipids (PUFAS), nucleobases and derivatives, organic acids and derivatives, piperidines, purines and derivatives and, purine nucleosides (Figure 4). Temporal variations in metabolite composition were observed throughout fermentation.

In quinoa tempeh, an increase in the relative abundance of amino acids and derivatives was observed on day 1, accompanied by a decrease in organic acids and derivatives. A similar trend was observed in tarwi tempeh at day 1; however, a decrease in hydroxy acids was also evident. In both substrates, amino acids and derivatives, along with organic acids, remained the predominant metabolite groups throughout the fermentation process.

Among the most abundant compounds within these groups were phenylalanine, valine, tyrosine, lactic acid, succinic acid, citric acid, and D-malic acid.

**Figure 5:**
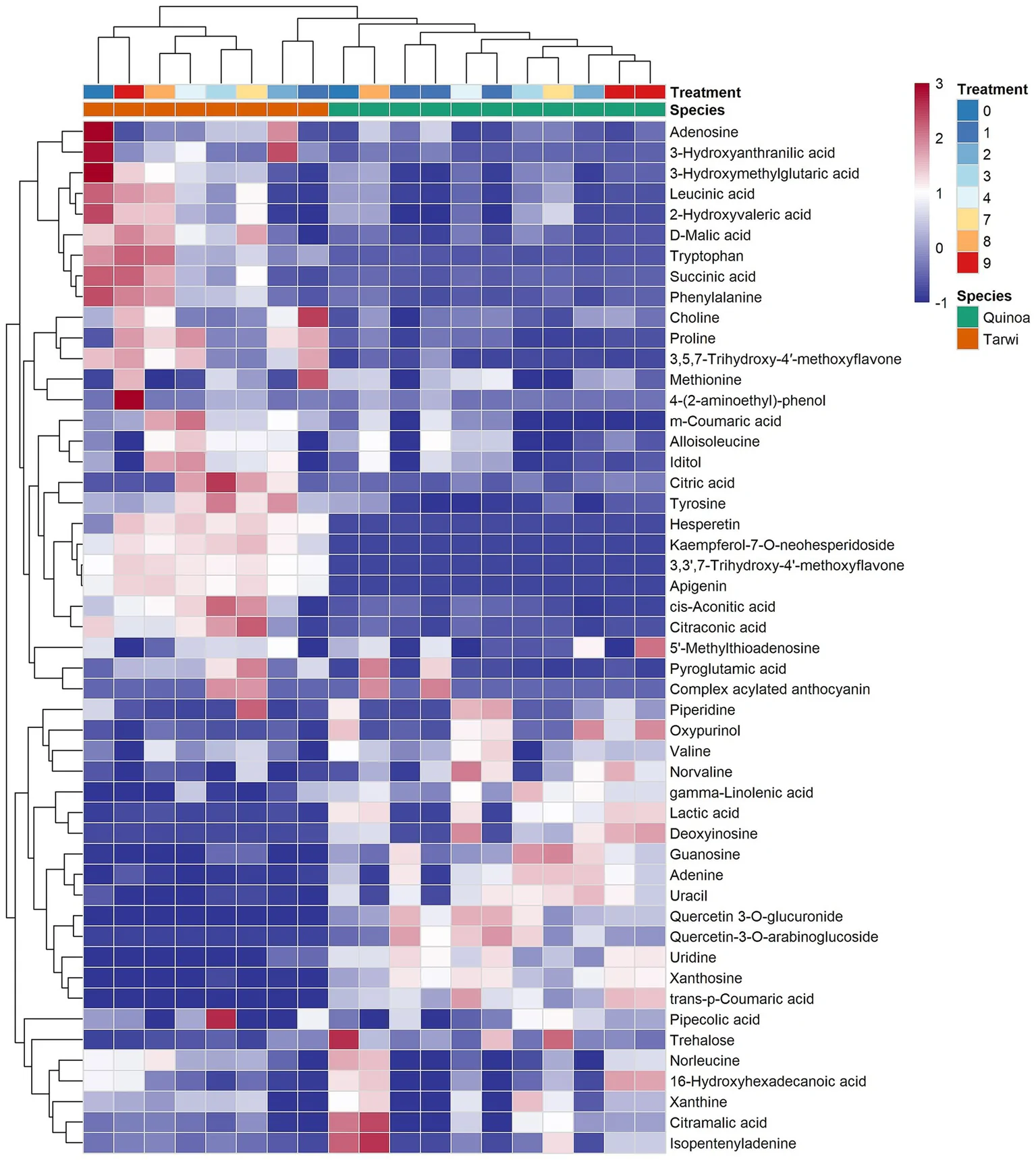
Hierarchical clustering heat map of metabolite-wise *z*-score normalized intensities for the 50 most abundant metabolites across fermentation time and species.

A total of 448 metabolites were annotated across all samples in the dataset, and the 50 most abundant and representative compounds were selected for heat map construction (Figure 5), revealing a clear substrate-dependent clustering pattern. Quinoa tempeh was primarily associated with metabolites such as lactic acid, *γ*-linolenic acid, and nucleoside- and nucleobase-related compounds, including xanthine, adenosine, guanosine, adenine, uracil, and isopentenyladenine, as well as organic acids such as citramalic acid. The prevalence of these compounds suggests an increase in nucleotide turnover and greater fermentative metabolic activity, associated with the production of acids derived from carbohydrate metabolism. In contrast, tarwi tempeh was characterized by stronger associations with metabolites related to amino acid metabolism and organic acid intermediates, including pyroglutamic acid, 3-hydroxymethylglutaric acid, 2-hydroxy-4-methylpentanoic acid, D-malic acid, and succinic acid. Additionally, several amino acids and related compounds—such as phenylalanine, tryptophan, tyrosine, proline, and leucinic acid—along with choline, were more prominently represented. This metabolite profile reflects an enhanced contribution of proteolysis and amino acid metabolism, consistent with the high protein content of tarwi and the increased proteolytic activity observed during fermentation.

**Figure 6:**
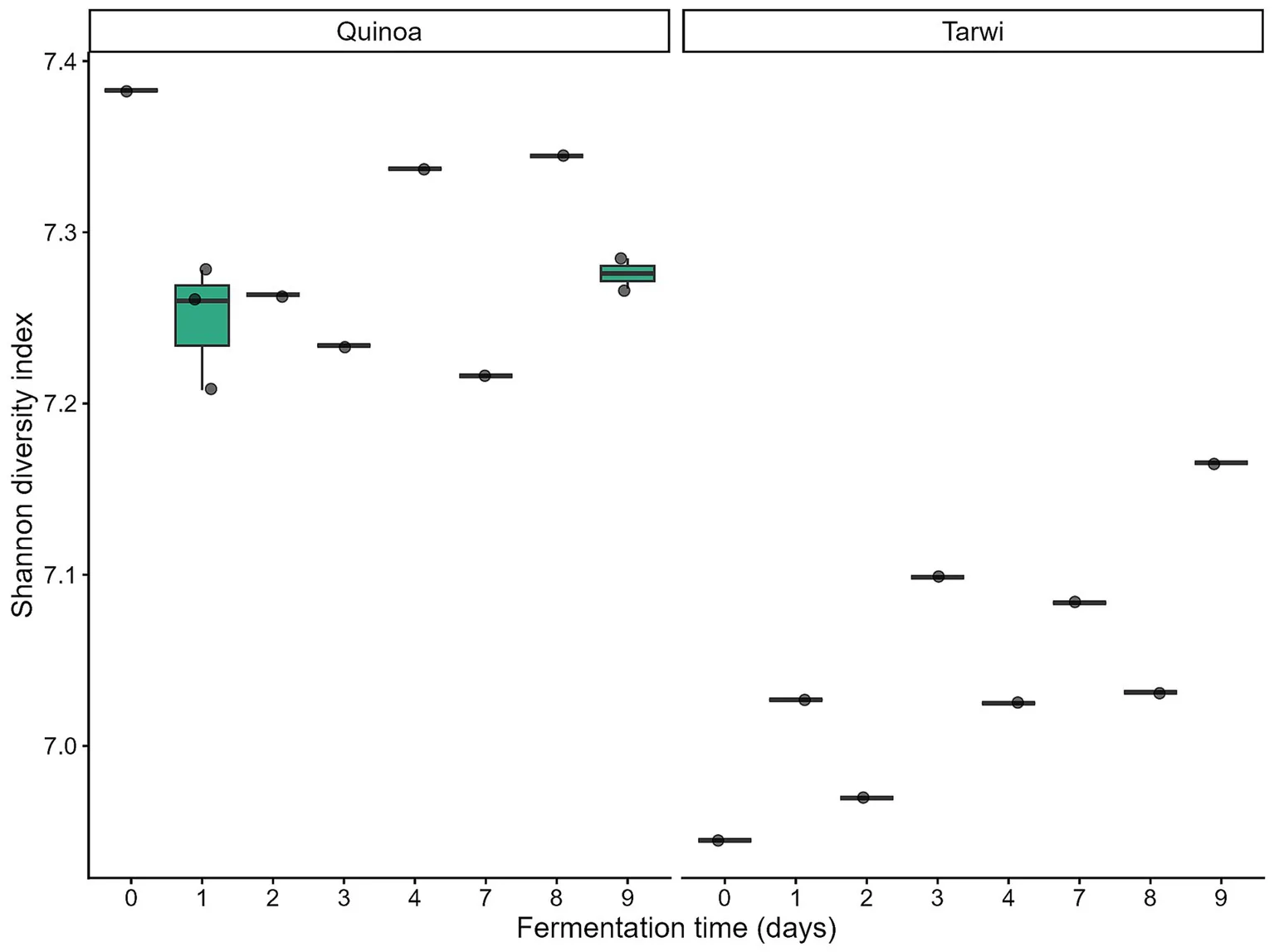
Shannon diversity index calculated from metabolite features with signal intensities greater than 200,000 counts.

Figure 6 presents the Shannon diversity index throughout the fermentation process. Overall, quinoa tempeh exhibited a more complex metabolite profile with a more even distribution of metabolite abundances, as reflected by its consistently higher H′ values. In contrast, tarwi tempeh displayed a profile dominated by a smaller number of metabolites, suggesting a more specialized and directed metabolism.

In both substrates, the Shannon index displayed a dynamic, non-linear pattern without a clear monotonic trend, reflecting the continuous processes of metabolite production and consumption during fermentation.

## 4 Discussion

### 4.1 Biomass

The fungal cell wall plays a fundamental role in maintaining cellular integrity, providing mechanical protection and preserving cell shape. It is composed of two main layers: an outer layer, which is more variable among fungal species and consists of proteins, pigments, and structural polysaccharides, and an inner layer, which is relatively conserved and primarily composed of *β*-glucans and chitin (31). Chitin is a linear homopolymer of β-(1,4)-linked N-acetylglucosamine units that forms extensive intra- and intermolecular hydrogen bonds, resulting in highly ordered, antiparallel polysaccharide structures analogous to cellulose (32). Due to its structural role in the fungal cell wall, chitin can be indirectly quantified by measuring the glucosamine content following hydrolysis. Therefore, glucosamine determination has been widely used as an indicator of fungal biomass and growth in fermented products such as tempeh (33).

Previous studies (34) have reported a progressive increase from 7.36 to 40.5 mg of glucosamine per g of tempeh from gars pea seeds between 8 and 32 h of fermentation. Although these values are superiors than those obtained in the present study for both quinoa (0.17–1.03 mg of glucosamine per g of tempeh) and tarwi (0.31–1.47 mg of glucosamine per g of tempeh), a similar increasing trend was observed. Notably, glucosamine accumulation was more pronounced in tarwi tempeh, where it continued to rise until 216 h of fermentation, suggesting sustained fungal growth and substrate consumption.

The high biomass values can be partly attributed to the glucosamine-based biomass determination method used in this study, as it can be influenced by complex agricultural substrates containing structural components, such as glycoproteins, glycosylated proteins, and cell wall polysaccharides, which can interfere with glucosamine quantification (23). In quinoa, the presence of glycosylated proteins has been reported (35), which is consistent with its high content of storage proteins such as globulins and albumins that are susceptible to glycosylation (36). Similarly, tarwi contains protein fractions including lectins and globulins (37, 38), which are known to interact with carbohydrates and may form complex structures within the seed matrix. These native components could contribute to an overestimation of glucosamine content, potentially explaining the high initial biomass values observed in both tempeh samples. Therefore, the interpretation of the results focuses primarily on the growth trends rather than on absolute biomass values, particularly at the initial sampling point. Nevertheless, direct measurements of fungal biomass or complementary biomarkers should be considered in future studies to further validate the observed growth kinetics.

Other study (39) reported a 2.48-fold increase in glucosamine concentration after 48 h of fermentation. In comparison, the present study showed a fourfold increase in quinoa tempeh and a 1.8-fold increase in tarwi tempeh relative to day 0, confirming that fungal growth occurred in both substrates, although with different kinetics.

### 4.2 Enzymatic activity

During tempeh fermentation, microorganisms of the genus *Rhizopus* produce a wide range of hydrolytic enzymes that degrade macromolecules present in the plant substrate into simpler and more bioavailable compounds. These enzymes include proteases, amylases, lipases, and phytases, which are involved in the hydrolysis of proteins, carbohydrates, lipids, and antinutritional factors. Therefore, enzymatic activity can be used as an indirect indicator of microbial growth (40).

Amylases are extracellular enzymes secreted into the surrounding medium, where they catalyze the breakdown of starch into mono- and disaccharides that can be utilized as carbon sources for microbial growth (41). The production of these enzymes is often substrate-induced; thus, their activity reflects both substrate composition and microbial adaptation. In this context, quinoa (var. Pasankalla) contains approximately 46% starch (42), whereas *Lupinus mutabilis* presents a much lower starch content (6–7%) (43). Based on this compositional difference, a higher amylase activity would be expected in quinoa tempeh. The availability and type of nutrients, especially nitrogen, have a significant effect on the growth and production of extracellular enzymes in *Rhizopus* spp. (44). Therefore, the higher protein content of tarwi could provide a more favorable nitrogen supply and a potentially lower carbon-to-nitrogen (C/N) ratio, which could promote fungal growth and the production of extracellular enzymes.

The relatively high amylase and protease activities observed in both samples at time zero are likely attributable to endogenous enzymes present in the raw materials rather than microbial activity. In quinoa, grains are known to contain intrinsic hydrolytic enzymes (45), with reported initial amylase activities of up to 8.9 U/mg in untreated or germinated seeds (46), these could have been active until they were inactivated in the cooking process prior to fermentation. Furthermore, pre-treatment steps such as soaking and washing, which resemble early stages of germination, may activate these endogenous enzymes (47). A similar effect can be expected in legumes such as tarwi, where hydration processes can promote enzymatic activation and the release of intracellular enzymes (48).

Previous studies have reported maximum amylase production at 168 h for *Rhizopus oryzae* grown on solid cassava waste and at 60 h for *Rhizopus oligosporus* grown on soybean substrate (25, 49). These findings are consistent with the present results, where maximum amylase activity in quinoa tempeh occurred on 48 h, while tarwi tempeh reached its highest activity on 168 h.

Regarding protease activity, previous studies have reported that during tempeh fermentation of *R.oligosporus* in beans, proteolytic activity initially decreases within the first 24 h due to the adaptation phase of *R. oligosporus*, followed by an increase as the microorganism enters the exponential growth phase, and a subsequent decline associated with the stationary and death phases (50). Similarly, it has been observed in Koro kratok fermentations when proteolytic activity becomes evident after 24 h, coinciding with visible mycelial growth, with optimal activity reported around 48 h (51, 52). These trends are consistent with the present study, in which protease activity increased after 48 h (2 days) in both quinoa and tarwi tempeh.

Notably, higher protease activity was observed in tarwi tempeh, which can be attributed to its elevated protein content (41–52%) (53, 54) compared to quinoa (20%) (42). This higher protein availability likely provides a greater substrate for proteolytic enzymes, thereby explaining the enhanced protease activity observed in tarwi-based tempeh.

### 4.3 Growth and enzyme production modelling

According to the results, the Gompertz model exhibited a higher coefficient of determination compared to the Logistic model, indicating a better fit for describing the growth of *Rhizopus oligosporus* on the evaluated substrates. This finding is consistent with previous studies that have successfully applied the Gompertz model to characterize the growth of filamentous fungi using almond hull and wheat bran as substrates (55, 56).

Differences in specific growth rates between tarwi and quinoa were also observed using the Gompertz model. These variations can be attributed to differences in substrate composition, which strongly influence fungal growth kinetics, affecting both maximum biomass and specific growth rate (57). Furthermore, the growth rates obtained in this study were lower than those reported in the literature (0.12–0.42 h^−1^) for filamentous fungi using coffee residues as substrate (58) highlighting that kinetic parameters are highly dependent on both the fungal strain and the substrate characteristics.

The *α* and *β* parameters of the Luedeking–Piret model represent growth-associated and non-growth-associated product formation, respectively. When α values exceed β values, enzyme production is primarily growth-associated (59). This behavior was observed in both tarwi and quinoa tempeh, suggesting that enzyme production is closely linked to fungal biomass formation. In filamentous fungi, increased mycelial density provides a larger surface area for enzyme secretion (60), which supports the observed relationship between growth and enzyme production. This effect was more pronounced in tarwi, where a denser mycelial network was observed. Previous studies have also demonstrated that the Luedeking–Piret model is suitable for describing product formation such as organic acids and enzymes in filamentous fungal systems (61, 62).

According to the Luedeking–Piret model, enzyme production in tarwi tempeh exhibited a better fit, as reflected by the higher goodness-of-fit statistics, indicating that enzyme synthesis was closely associated with fungal growth. This finding is consistent with the greater mycelial development observed in tarwi and its higher proteolytic activity. In contrast, quinoa showed a poorer fit, particularly for amylase activity (*R*^2^ = 0.05), suggesting that enzyme production was less directly linked to biomass accumulation and was likely influenced by additional physiological processes related to substrate composition and carbohydrate metabolism. These distinct patterns of enzyme production likely contributed to the substrate-specific modifications of the food matrix, thereby influencing both the textural properties and the metabolomic profiles observed during fermentation.

### 4.4 Texture profile analysis (TPA)

Hardness values ranging from 8.29 to 36.82 N have been reported for different tempeh samples (Soybean, chickpea, green lentil, red lentil, white bean, black bean and broad bean), with similar ranges observed for fava bean tempeh (8.5–26.3 N) (63, 64). These values are markedly lower than those obtained in the present study for quinoa (78–154.14 N) and tarwi (42.92–82.31 N) tempeh, suggesting that differences in raw material composition and structural properties may significantly influence textural development during fermentation.

The fragility of tarwi tempeh (14.02–44.08 N) fell within the previously reported range (4.71–34.10 N) (63), except for the sample fermented for 2 days, which exhibited the highest value (44.08 N). Therefore, tarwi tempeh required a greater force to initiate structural fracture or fragmentation (65) than quinoa tempeh, in which no fragility was detected, suggesting the absence of a distinct fracture point during compression, likely due to less extensive mycelial development.

Cohesiveness and springiness values obtained in this study (0.1–0.15 and 0.22–0.35 respectively) were consistent with those reported in previous works using broad beans, soybeans, chickpeas, lentils, and beans as substrates (63, 64, 66). Notably, quinoa tempeh on day 3 exhibited higher gumminess (19.76 N) than previously reported values (0.62–15.56 N), while the remaining samples fell within the reported range (63). A similar trend was observed for chewiness, with quinoa tempeh on day 3 (6.35 N) exceeding previously reported values (0.8–5.24 N), whereas other quinoa and tarwi tempeh samples showed comparable results (63, 64).

The increase in hardness and fragility observed in tempeh can be attributed to the growth of *Rhizopus oligosporus* mycelium, which forms a dense and resistant network within the substrate (67). This mycelial structure acts as a binding matrix, holding the grains together and promoting the formation of cohesive textures (68), which explains the progressive increase in cohesiveness over time observed in the results.

The subsequent reduction in hardness in quinoa tempeh can be explained by the enzymatic activity of amylases produced by *R. oligosporus*, which hydrolyze starch and reduce structural firmness (68). In addition, prolonged fermentation, such as that applied in this study, has been reported to significantly decrease tempeh hardness (67). In quinoa, starch is likely gelatinized during the initial cooking process, making it more susceptible to enzymatic degradation. Consequently, increased amylolytic activity promotes the breakdown of starch granules into smaller fragments, along with the degradation of cell wall components, leading to a weakened structure (69).

In the case of tarwi tempeh, the reduction in hardness and fragility after 48 h is mainly associated with its higher protein content and the corresponding increase in proteolytic activity compared to quinoa. The progressive breakdown of complex structural components through enzymatic activity leads to the formation of a softer and less resistant matrix (18, 70). In addition, excessive fungal growth during prolonged fermentation promotes structural deterioration beyond the optimal fermentation period, resulting in a decline in texture parameters (67). Based on the texture profile analysis, the optimal fermentation time was approximately 48 h for tarwi tempeh and 72 h for quinoa tempeh.

With respect to adhesiveness, previous studies have reported low negative values, reflecting limited stickiness in conventional tempeh products (63, 64, 66). In contrast, only quinoa tempeh on day 1 showed comparable behavior, whereas the remaining quinoa and tarwi samples exhibited higher adhesiveness, suggesting enhanced interfacial interactions and surface properties.

Adhesiveness is influenced by soluble compounds, such as polysaccharides, sugars, and peptides, generated during fermentation through the hydrolysis of starch and other macromolecules (71). The higher adhesiveness observed during the intermediate stages of fermentation likely reflects the accumulation of these hydrolysis products, whereas its subsequent decline is consistent with their continued degradation during prolonged fermentation, reducing water retention and surface interactions (69).

Springiness showed contrasting behavior between studied substrates. In tarwi tempeh, the decrease in springiness can be attributed to proteolytic activity, which breaks down protein structures into smaller fragments, resulting in a softer and less elastic matrix (72, 73), reducing their capacity for elastic recovery. In contrast, quinoa tempeh exhibited an increase in springiness, which could be attributed to structural reorganization during fermentation associated with amylolytic activity. Given quinoa’s high starch content, enzymatic hydrolysis of starch by amylases could alter the polysaccharide matrix and macromolecular interactions. Controlled enzymatic hydrolysis has been reported to improve structural properties (74), promoting the formation of composite networks of proteins and polysaccharides that enhance elasticity (75).

Texture evaluation is important because it contributes to fundamental properties associated with consumer acceptance and overall food quality (76). As previously discussed, an adequate fermentation time promotes desirable texture attributes such as hardness, springiness, cohesiveness, and gumminess, which are characteristic of high-quality tempeh products (63). In contrast, excessive fermentation results in reductions in hardness and cohesiveness, likely due to excessive fungal growth and the progressive degradation of structural macromolecules (67, 68, 70), leading to softer and less stable textures. Therefore, the observed textural changes indicate that fungal growth and enzymatic activity play a key role in defining the organoleptic characteristics of quinoa- and tarwi-based tempeh, highlighting their potential as fermented foods derived from Andean grains.

### 4.5 Metabolomics analysis

Principal component analysis (PCA) revealed a clear separation between quinoa and tarwi samples along the principal components, indicating that substrate composition is the main factor driving differences in metabolite profiles because they belong to different taxonomic families (77, 78), also observed in Supplementary Figure S1.

Regarding the effect of fermentation time, quinoa samples exhibited tighter clustering, suggesting a more homogeneous and stable metabolite profile throughout the process. In contrast, tarwi samples were more widely dispersed, indicating greater temporal variability in metabolic composition. This behavior may be associated with enhanced fungal growth in tarwi, as evidenced by higher biomass accumulation, which occurs due to enzymatic activity (79, 80), thereby contributing to a broader distribution of metabolites over time.

Temporal changes in metabolite classes revealed an inverse relationship between amino acids and their derivatives and organic acids and their derivatives, reflecting their interconnection through central metabolic pathways (81). During the early stage of fermentation, organic acid production remained limited as Rhizopus oligosporus adapted to the substrate, while pre-existing organic acids may have been consumed as carbon sources to sustain fungal growth (82, 83).

The initial increase in amino acids observed on day 1 can be attributed to proteolytic activity, which promotes the release of amino acids, peptides, and related compounds (84). In quinoa tempeh, a rapid decrease in amino acids accompanied by an increase in organic acids up to day 3 suggests a predominance of carbohydrate metabolism, likely driven by higher amylase activity, where sugars are preferentially utilized for organic acid production (85). Subsequently, the alternating increase and decrease of both amino acids and organic acids may reflect competition for shared metabolic intermediates, indicating dynamic shifts in metabolic pathways as fermentation progresses (81).

Quinoa tempeh was primarily associated with metabolites such as lactic acid, *γ*-linolenic acid, and nucleoside-related compounds, including xanthosine, adenosine, and guanosine. The prevalence of these compounds throughout fermentation suggests intensified carbohydrate metabolism and active cellular turnover which generates soluble sugars that serve as carbon sources for the growth of *Rhizopus oligosporus* (84). In particular, the accumulation of lactic acid reflects an active fermentative metabolism linked to energy production (44).

In contrast, tarwi tempeh showed stronger associations with metabolites related to amino acid metabolism, including phenylalanine and pyroglutamic acid, as well as tricarboxylic acid (TCA) intermediates such as succinic and D-malic acids. This pattern is consistent with the higher protein content of tarwi and the greater proteolytic activity observed, which promotes the release and subsequent transformation of amino acids. During fermentation, *Rhizopus oligosporus* utilizes available carbohydrates for growth while simultaneously producing proteases that hydrolyze proteins into peptides and free amino acids (84). The temporal variation in amino acid levels further suggests a dynamic balance between their production and utilization, which is characteristic of prolonged fermentation processes (86).

Overall, the heat map reveals a clear metabolic distinction between the substrates. Quinoa fermentation is characterized by carbohydrate-driven metabolism and nucleotide turnover, whereas tarwi fermentation is dominated by proteolysis and amino acid-derived metabolic pathways. These findings are consistent with the enzymatic activity patterns and PCA results, reinforcing the central role of substrate composition in shaping metabolomic profiles during tempeh fermentation.

The Shannon diversity index further supports these observations. Quinoa exhibited greater metabolic diversity, particularly on day 1, likely reflecting the initial release of a wide range of soluble compounds following hydration and early enzymatic activity, especially amylolysis (44). As fermentation progressed, the gradual decline in diversity suggests selective metabolite consumption and conversion into a smaller number of dominant compounds, consistent with metabolic specialization (40). In both substrates, the non-linear behavior of the Shannon index indicates a dynamic equilibrium between metabolite production, transformation, and consumption, driven by temporal changes in enzymatic activity and fungal growth (84). This behavior is characteristic of fermentation systems, where metabolite concentrations fluctuate due to ongoing biochemical transformations (87), and aligns with the patterns observed in both the heat map and PCA analyses.

The metabolomic profile demonstrated extensive biochemical transformations during fermentation that were largely dependent on substrate composition. Tarwi fermentation was characterized by amino acid-related metabolites, consistent with its higher proteolytic activity and the extensive degradation of proteinaceous compounds during fermentation (88). In contrast, quinoa fermentation showed a predominance of metabolites associated with carbohydrate metabolism and nucleotide turnover, including organic acids and nucleoside-derived compounds, reflecting active fermentative metabolism (66, 89). These metabolomic patterns were consistent with the enzyme activity and texture results, confirming that substrate composition plays a key role in governing the biochemical transformations occurring during tempeh fermentation. Although these metabolic changes may influence the nutritional characteristics of tempeh, their biological significance should be confirmed through targeted metabolomic analyses and complementary *in vitro* or *in vivo* studies.

Although commercial tempeh production is typically completed within 24–48 h (17), the extended fermentation period evaluated in this study enabled a comprehensive assessment of fungal growth, enzyme production, textural changes, and metabolomic dynamics throughout the fermentation process. Nevertheless, from a technological perspective, the results indicate that 48–72 h represents the optimal fermentation period, providing the best balance between mycelial development, texture, and biochemical transformations.

## 5 Conclusion

This study demonstrated that substrate composition plays a decisive role in shaping growth kinetics, enzymatic activity, texture, and metabolomic profiles during tempeh-type fermentation by *Rhizopus oligosporus*. The Gompertz model provided the best fit for fungal growth, confirming its suitability for describing biomass development, particularly in tarwi. Enzyme production, indirectly evaluated through enzymatic activity, was predominantly growth-associated, as indicated by the Luedeking–Piret model, with a more pronounced effect observed in tarwi.

Tarwi tempeh exhibited greater proteolytic activity and a metabolomic profile dominated by amino acid-related compounds such as phenylalanine, tryptophan, choline, and pyroglutamic acid, reflecting intensified proteolysis and the potential generation of bioactive and nutritionally relevant compounds. In contrast, quinoa tempeh showed a stronger amylolytic influence, greater metabolic diversity, and a higher abundance of organic acids and nucleoside-related compounds such as lactic acid, *γ*-linolenic acid, xanthine, adenosine, guanosine, adenine, and citramalic acid, indicating a metabolism more closely associated with carbohydrate utilization and energy production.

Textural properties, including hardness, cohesiveness, and springiness, were closely linked to fungal growth and enzymatic activity, particularly through mycelial network development and the progressive degradation of macromolecules, observing better characteristics between 48 and 72 h of fermentation, reducing its properties in prolonged fermentation times.

Overall, the integration of kinetic modeling, enzymatic activity, texture evaluation, and metabolomic analysis provides a comprehensive understanding of the fermentation dynamics and highlights the potential of tarwi and quinoa as alternative substrates for the development of tempeh-type products with promising highly nutritional and functional properties.

The main limitations of this study are that biomass was estimated indirectly through glucosamine quantification and that the metabolomic findings were not biologically validated. Therefore, future studies should validate the biological relevance of the identified metabolites through *in vitro* and *in vivo* assays, optimize fermentation conditions at the pilot scale, and evaluate the sensory acceptance and shelf life of tempeh-type products derived from Andean crops.

## Data Availability

The raw data supporting the conclusions of this article will be made available by the authors, without undue reservation.

## Supplementary material

SUPPLEMENTARY FIGURE S1 PCA biplot showing the contribution of the ten metabolites with the highest loadings to sample discrimination between quinoa and tarwi. Metabolites are colored according to their chemical class.
